# Mitigating the Stigma of Mental Illness: The Impact of Story-Telling in the Black Community

**DOI:** 10.3390/ijerph21111473

**Published:** 2024-11-06

**Authors:** Kyaien O. Conner, Daniel K. Abusuampeh, Kristin Kosyluk, Jennifer T. Tran, Denise Davis-Cotton, Angela M. Hill, Alexus P. Brown

**Affiliations:** 1 School of Social Work, University of Pittsburgh, 4200 Fifth Ave, Pittsburgh, PA 15260, USA; dka18@pitt.edu (D.K.A.); browna43@duq.edu (A.P.B.); 2 Department of Mental Health, Law & Policy, College of Behavioral & Community Sciences, University of South Florida, 4204 E. Fowler Ave, Tampa, FL 33612, USA; kkosyluk@usf.edu; 3 Department of Family and Community Health, School of Nursing, University of Pennsylvania, 418 Curie Blvd, Philadelphia, PA 19104, USA; jtgtran@nursing.upenn.edu; 4 Florida Center for PAInT, University of South Florida, 8350 N. Tamiami Trail, Sarasota, FL 34243, USA; ddaviscotton@usf.edu; 5 Taneja College of Pharmacy, University of South Florida, 12901 Bruce B. Downs Blvd, Tampa, FL 33612, USA; ahill2@usf.edu

**Keywords:** stigma, mental health, treatment, recovery

## Abstract

Racial/ethnic minorities, including the Black community, experience stigma as a significant barrier to mental health care, with fears of being devalued or discriminated against deterring individuals from seeking help. Racial stigma further exacerbates mental health issues and negatively influences perceptions of service utilization. To address this, our research team partnered with a national non-profit storytelling organization to develop and evaluate a virtual narrative storytelling intervention series that amplifies the voices and experiences of Black Americans living with mental illness and addiction. We randomly assigned 193 participants to either the intervention (n = 102) or an active control condition (n = 91) and used a pre–post survey design to assess the changes in the outcome variables. Contrary to our hypothesis, there were no race-based interactions; instead, the results show significant reductions in public stigma and perceived discrimination and increased positive attitudes toward seeking treatment universally among all the intervention participants. This study provided preliminary evidence that a virtual storytelling intervention is instrumental across varied demographic cohorts, transcending potential cultural barriers in the discourse and understanding of mental health to effectively mitigate stigma and improve attitudes toward mental health treatment.

## 1. Introduction

Mental health disorders continue to be identified as one of the leading causes of disability worldwide [1]. They are experienced by nearly 44 million US adults (1 in 5) every year [2]. Black Americans are more likely than white Americans to report persistent symptoms of emotional distress and suffer from a higher rate of psychological difficulties [3,4]. The higher incidence of mental distress in the Black community has been associated with prejudice and racism experienced in daily life, the lack of access to appropriate and culturally responsive mental health care, and the legacy of historical trauma [5,6,7,8,9,10,11,12]. Issues related to economic insecurity and disproportionate experiences with violence, criminal injustice, and child welfare further compound these mental health disparities [13,14].

Despite a greater recognition of the importance of mental health and the benefits of mental health treatment, we continue to experience a significant treatment gap for mental disorders in the United States [15]. While this speaks to a significant challenge in the United States, some groups are more likely to experience delays or a lack of service utilization than others. Despite research suggesting Black Americans are 20% more likely to experience mental illness, only one in three Black adults who need mental health care receive it each year, about half that of non-Hispanic White Americans [16]. Untreated mental illness leads to various adverse outcomes for the individual, the healthcare system, and society, thus highlighting the importance of addressing barriers to mental health service utilization. Research identified several systemic barriers to treatment engagement for Black Americans, including family privacy, lack of knowledge regarding available treatments, denial of mental health problems, and concerns about medications and other treatment, as well as treatment costs, insurance, and transportation challenges [17,18]. Negative attitudes about mental health treatment were identified as a salient barrier to treatment engagement among Black Americans [19,20]. Among the multiple explanations, stigma has been recognized as one of the most prevailing barriers to mental health care in the Black community [21,22,23,24].

Stigma is experienced as perceived public stigma (e.g., fear of being treated differently, de-valued, or discriminated against by others due to having a mental illness) and internalized stigma (e.g., feelings of shame, guilt, and diminished self-esteem), which deters people from seeking mental health care [25,26,27]. Unfortunately, research indicates that mental illness stigma is amplified in the Black community [22,28,29,30]. A recent survey suggests that 63% of African Americans believe a mental health condition is a personal sign of weakness and that seeking treatment is a last resort or not an option [31]. These beliefs, often culturally specific and based upon a foundation of cultural norms and mistrust of health care and healthcare providers, further deter Black Americans from being open about having a mental health challenge and seeking professional mental health services when needed [22,30]. Strategies to reduce stigma and improve knowledge and attitudes about seeking treatment are urgently needed.

Evidence-based stigma reduction approaches include education and contact interventions, with research supporting contact-based approaches (i.e., personal interaction with individuals living with a mental illness) as superior at changing mental illness stigma among adults [30,32]. Contact-based interactions involve individuals with mental illnesses sharing their stories of lived experiences. Research further suggests that people are the most responsive to contact-based interventions when the messages are targeted toward specific populations (e.g., having people from the targeted population deliver the message) and when it involves contact with peers who have experienced mental health problems themselves [33,34]. Racial differences in both experiences of stigma and receptivity of potential audiences make the production and dissemination of Black stories utterly crucial.

Virtual interventions have increased exponentially since the COVID-19 pandemic. Stay-at-home orders and concerns about health and safety led to the necessity of enhancing telehealth and virtual health models of intervention. These strategies can be particularly crucial for racial and ethnic minorities who are facing physical barriers to care, as well as the stigma associated with seeking help. Research suggests that virtual interventions (e.g., media-based contact) involving education and contact can reduce the perceptions of stigma and improve attitudes toward mental illness and mental health treatment [35,36,37]. To address this need, we partnered with a national non-profit storytelling organization to develop a storytelling-based stigma reduction intervention to amplify the Black and Brown voices of individuals living with mental health challenges to reduce the stigma among this population and all who viewed this intervention and to enhance anti-racism and positive attitudes about mental health treatment.

### The Project of This Is My Brave: Stories from the Black Community

This Is My Brave (TIMB) is a national non-profit organization that aims to end mental illness stigma. TIMB provides a platform for individuals with mental illness to share their stories of recovery and hope on stage through creative means (e.g., poetry, song, dance, monologue). Research shows that TIMB effectively reduces public stigma, reduces discrimination, improves beliefs about recovery from mental illness, and improves attitudes toward treatment seeking [38,39]. Due to the need for a culturally responsive mental illness stigma reduction program, a special edition show was created, “This Is My Brave: Stories from the Black Community” (TIMB: SBC). The development of TIMB: SBC involves storytellers working with social workers or other mental health professionals and the TIMB team to craft their story in meaningful ways. This process involves multiple meetings with the TIMB team and clinicians with the storytellers in a group format bi-weekly over a course of three months to practice developing and sharing their story. It is this process of story development that makes the TIMB intervention incredibly effective, as the storytellers learn how to develop and engage the audience in the narrative. Additional detail about the development of TIMB is published elsewhere [40]. Please use this link to take you to an online platform to view this series: https://thisismybrave.org/announcing-stories-from-the-black-community/ (accessed on 1 July 2024).

In a pilot study, we found that TIMB: SBC effectively reduced public stigma and increased attitudes toward treatment seeking and anti-racist attitudes toward the Black community. We also found interaction effects, where the intervention more saliently impacted the outcomes for Black viewers of the intervention [40]. While this pilot study provided initial findings on the effectiveness of TIMB: SBC, we did not have an active control condition, impacting our ability to make causal statements about the impact of this intervention. This study built upon this pilot work and this paper presents the findings of a randomized controlled trial to investigate the effectiveness of stories from the Black community in reducing mental illness stigma and increasing anti-racist attitudes in a nationwide sample as compared with individuals who received an educational video unrelated to mental health. We hypothesized that viewers who received the intervention would have reductions in stigma and improvements in attitudes about seeking treatment and anti-racism as compared with the individuals who received the control condition. We also hypothesized that Black viewers would be more saliently impacted by the intervention than White viewers.

## 2. Materials and Methods

### 2.1. Sample and Study Procedures

The participants were recruited nationally through the platform Prolific, a crowd-working platform suited for the needs of the scientific community of researchers [41]. Prolific allows for a more diverse population that is naïve to experimental research [42]. The specific demographics of these participant population are shown in Table 1 (seen on page 5-6 of the document). Two separate population samples were recruited: (1) individuals who identified as Black or African American and (2) who identified as non-Hispanic White. Participants were randomized into one of two conditions: (a) the control condition, where participants watched a nature video unrelated to mental health (about 30 min), or (b) the intervention, where participants watched a shortened TIMB: SBC (about 30 min). We randomized the participants using a random numbers algorithm that was built into our Prolific platform. The participants could not skip through the video and were asked to provide a short description afterward. Participants who did not answer this attention check question were removed from the analyses. The participants were balanced across the video conditions based on race/ethnicity and based on sex (male and female). All participants received USD 15 through Prolific for their time and completion of this study. This study was submitted to our university’s institutional review board; however, it was deemed exempt due to its minimal risk.

### 2.2. Measures

The measures in this current study were identical to those in our open-trial pilot study [40]. We chose to utilize the same measures to ensure we could compare and contrast the current study findings with previous open-trial research. The measures chosen were also some of the most widely utilized and studies measures of the constructs we have chosen to observe. The measures included the Attribution Questionnaire (AQ-9) [25], the Perceived Devaluation–Discrimination Scale (PDDS) [43], the Social Distance Scale (SDS) [44], the Semantic Differential: Similar–Different scale [45], the Attitudes Toward Mental Health Treatment Scale (ATMHT) [46] and an Anti-Racism Scale.

### 2.3. Personal Stigma

Personal stigma is an individual’s attitudes, stereotypes, prejudices, and behaviors toward individuals living with a mental health condition. Personal stigma was measured using the Attribution Questionnaire (AQ-9), which has good internal consistency (α = 0.73), test–retest reliability (r = 0.73), and construct validity [25]. The vignette preceding the measures speaks about Harry, who is living with schizophrenia, and is followed by nine questions that are answered on a nine-point Likert scale. An example question is, “How dangerous would you feel Harry is?” Higher scores on the AQ-9 indicate higher levels of personal stigma.

### 2.4. Perceived Stigma

Perceived stigma refers to one’s perceptions of how stigmatizing their community is toward a person with a mental health condition. Perceived stigma was measured using the Perceived Devaluation–Discrimination Scale (PDDS) [43]. The PDDS is a 12-item instrument asking participants to indicate how much they agree with statements on their perceptions of their community toward individuals with mental health conditions. An example item reads, “Most people feel that entering a psychiatric hospital is a sign of a personal failure”. Items are responded to on a six-point Likert scale (1 = strongly agree and 6 = strongly disagree). Higher scores on the PDDS represent more significantly perceived public stigma.

### 2.5. Discrimination

Discrimination is the unjust or prejudicial behavior or treatment toward individuals with a marginalized identity, in this case, individuals living with a mental health concern. Discrimination was measured using the Social Distance Scale [44], as desired social distance is a proxy for discrimination [47,48,49,50,51,52,53,54,55,56,57]. The SDS measures seven items with good internal consistency (α = 0.75) and validity [49]. An example item reads, “How would you feel about renting a room in your home to a person with severe mental illness?” Participants rate items on a 3-point willingness scale (0 = definitely willing and 3 = definitely unwilling). Items are summed to create a total score for social distance, with higher scores indicating higher desired social distance or discrimination.

### 2.6. Difference

According to Modified Labeling Theory (MLT), one of the initial steps in the stigma process is the identification of traits that separate the stigmatized from the stigmatizer (out-group vs. in-group), resulting in a separation of us vs. them, adverse stereotyping emotional reactions, and behaviors toward members of the stigmatized group [50]. Measures of difference are used as a proxy for stigma. In the Semantic Differential: Similar–Different scale [45], the vignette before the scale states, “Eric is a 30-year-old single man with schizophrenia. Sometimes, he hears voices and becomes upset. He lives alone in an apartment and works as a clerk at a large law firm. He had been hospitalized six times because of his illness”. Participants respond on a nine-point agreement scale for three items, with higher scores indicating a higher perceived difference.

### 2.7. Attitudes Toward Mental Health Treatment Seeking

Attitudes about seeking mental health services were assessed utilizing the Attitudes Toward Seeking Mental Health Treatment Scale (ATMHT) [46]. The ATMHT comprises 20 items intended to reflect positive or negative attitudes toward professional mental health treatment and is a modified version of the 29-item Attitudes Toward Seeking Professional Psychological Help Scale (ATSPPHS) [51]. The ATMHT scale asks about the extent of agreement on a four-point Likert scale, ranging from strongly disagree (1) to strongly agree (4), with statements such as “Most mental health professionals have negative beliefs about the mentally ill”. High scores on the ATMHT (scores range from 20–80) reflect more positive attitudes about seeking mental health treatment.

### 2.8. Anti-Racism

Anti-racism was assessed using 12 questions that asked participants to better understand their anti-racism beliefs, which focused on Black Americans. The questions ask about the extent of agreement on a four-point Likert scale, ranging from strongly disagree (1) to strongly agree (4). Items included statements such as “The reason that mental health disparities exist within the Black community is that Black people do not work hard enough to improve their health outcomes” and “Racism worsens mental health stigma within the Black community”. High scores on these items (ranging from 20–80) reflect more positive anti-racism beliefs.

### 2.9. Data Analysis

All analyses were performed in SPSS Version 28 (IBM, Armonk, United States) [52]. Table 2 summarizes the means and standard deviations of each measure based on the conditions at the pre- and post-test, along with Cronbach’s alphas for each measure obtained from this sample. Descriptive statistics were used to describe the sample. Chi-square and t-tests were used to assess for significant differences between groups by condition based on demographic characteristics. There were no statistically significant differences between the condition groups regarding demographic variables.

A three-way interaction of time (pre and post) by condition (experimental and condition) by race (Black/African American and non-Hispanic White) using mixed repeated measure ANOVAs were used to examine the differences between the variables (personal stigma, perceived stigma, anti-racism, attitudes toward treatment seeking, discrimination, and difference).

## 3. Results

### 3.1. Participants

One hundred ninety-three participants completed the survey (102 control and 91 TIMB condition), with 58.5% identifying as Black/African American and 41.5% identifying as non-Hispanic White. The participants had an average age of 33.59 (SD = 11.21). The majority of the sample was single and had completed some college or had a bachelor’s degree. There were similar percentages of male and female respondents. Importantly, approximately 40% of the sample had a previously diagnosed mental health condition. Table 1 provides further detailed demographic information for participants based on the condition (TIMB or control).

### 3.2. Differences Between Conditions

To examine the effectiveness of the intervention compared with a control condition, we examined the two-way interactions. There was a significant two-way interaction of time condition condition for personal stigma (*F*(1,191) =6.62, *p* = 0.01), difference (*F*(1,191) = 24.38, *p* < 0.001), discrimination (*F*(1,191) = 3.98, *p* = 0.05), attitudes toward mental health treatment (*F*(1,191) = 10.39, *p* = 0.001), beneficial attitudes toward mental health treatment (*F*(1,191) = 7.53, *p* = 0.01), and pessimistic attitudes toward mental health treatment (*F*(1,119) = 5.19, *p* = 0.02).

**Figure 1:**
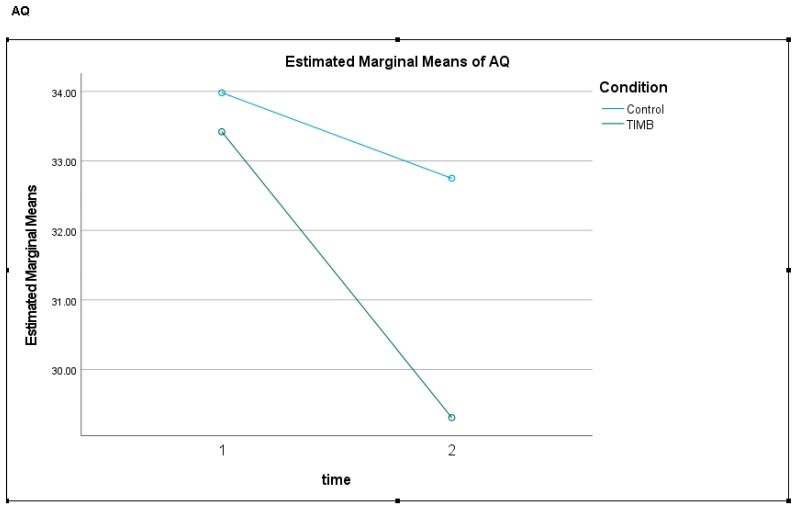
Graph of public stigma for participants in the control condition and intervention condition across time.

The participants who viewed the intervention (TIMB: SBC) showed a significant decrease from pre- to post-intervention in personal stigma; however, the participants in the control condition also showed a reduction in personal stigma across time (*p* = 0.038). However, the participants who viewed the intervention significantly decreased their personal stigma compared with the control condition (see Figure 1). Furthermore, the participants who viewed the intervention showed a significant decrease in discrimination across time compared with the participants in the control condition. Overall, there were decreases in personal stigma and discrimination for the participants who viewed the intervention.

The participants who viewed the intervention (TIMB: SBC) showed a significant improvement from pre- to post-intervention in attitudes toward mental health treatment and beneficial attitudes toward treatment. Unexpectedly, the participants in the intervention condition also experienced an increase in pessimistic attitudes compared with the participants in the control condition. The participants in the control condition showed no significant change in attitudes toward treatment (total, beneficial, or pessimistic) from pre- to post-intervention, indicating the effectiveness of the control condition in comparison. Table 2 provides information on the two-way interaction of time X condition and the means of the dependent measures based on the condition across time. This table highlights the significant differences in the variable’s personal stigma, discrimination, perceived difference, and attitudes toward seeking mental health treatment between the TIMB condition and the control group. Please see Appendix A Table A1 for *t*-test results of initial scores in Black versus White participants across variables.

## 4. Discussion

This investigation afforded crucial insights into the potential impact of anti-stigma and anti-racism interventions like “This Is My Brave: Stories from the Black Community” (TIMB: SBC) on stigma and mental health-related attitudes and beliefs. Notably, significant decreases in personal stigma and discrimination and augmentations in attitudes toward mental health treatment were documented among the participants subjected to the intervention (TIMB: SBC) in comparison with those in the control condition. Contrary to our hypothesis, the general impact of the intervention did not display significant differences in outcomes between the Black and non-Hispanic, White participants. However, this highlights the intervention’s widespread utility across varied racial/ethnic groups.

Racism, recognized as a public health crisis, exacerbates health disparities for Black Americans, particularly in mental health [53]. Historical and structural racism contributes to persistent socioeconomic inequalities, increased exposure to environmental stress, violence, and systemic barriers to accessing quality healthcare [54]. These factors amplify the mental health burden in Black communities, creating a dual challenge of stigma and insufficient care. By addressing both cultural norms and personal biases that deter help seeking, this intervention contributes to broader public health efforts aimed at mitigating the mental health treatment gap for all individuals, particularly racial and ethnic minorities.

The implications for public health research and practice are many, particularly given the documented efficacy of TIMB: SBC in mitigating stigma and enhancing treatment-seeking attitudes, aligning with previous research findings [38,39]. First, considering the current study observed a significant improvement in attitudes toward mental health treatment, these findings suggest that public health practitioners engaged in mental health services can employ creative, narrative-based interventions to foster conducive environments where individuals living with mental health conditions feel understood, respected, and less judged. Practitioners can work with TIMB to develop stories that are unique to the communities they work with, or they can utilize pre-existing narratives that have been empirically tested, like TIMB: SBC, which can be accessed for free online. Utilizing lived experience stories, like in TIMB: SBC, is integral in resonating with individuals personally, breaking down preconceived stereotypes, and building empathetic understandings of mental health challenges.

Second, this intervention was shown to significantly mitigate both perceived and personal stigma. The stigma surrounding mental illness, especially internalized or personal stigma, is a well-documented public health issue that prevents individuals from seeking care, leading to worsened health outcomes, increased healthcare costs, and greater societal burden [25]. Research shows that untreated mental illness is associated with a higher risk for physical health issues, increased morbidity, and greater healthcare resource utilization, emphasizing the importance of timely and accessible care [55]. Stigma, thus, functions as a barrier to public health, as it impedes access to necessary mental health services, particularly for Black individuals, who already face disproportionately high levels of psychological distress due to the compounded effects of racism and discrimination [56,57]. This study provided preliminary evidence that virtual, storytelling interventions can be effective at addressing this public health challenge.

Despite TIMB: SBC’s evidenced efficacy in curbing stigma and enhancing mental health treatment attitudes, the intervention also unexpectedly increased the pessimistic attitudes toward mental health treatment. While we did not anticipate this finding, it may be explained by storytellers sharing some of their negative experiences seeking effective and culturally sensitive care. While the participants shared their stories of success and recovery, which likely enhanced the viewers positive attitudes, they also shared some of their challenges and experiences of racism or microaggressions when seeking treatment, which may have impacted viewers more pessimistic attitudes about treatment. Overall, the total attitude scores were improved; however, this finding requires additional research and explanation to better understand the unanticipated consequences of implementing anti-stigma initiatives, echoing earlier concerns about the potential unintentional effects of mental illness anti-stigma campaigns [32].

Furthermore, the absence of distinct differences in the intervention’s efficacy across racial/ethnic lines challenges previously documented studies that found that racial and ethnic minorities experienced greater reductions in stigma and enhanced mental health treatment attitudes post-TIMB: SBC intervention [40]. Race, ethnicity, and mental health are complex and there is a myriad of other factors that could have impacted this result. For example, the average age of the study participants (33 years) aligned with the age range of our storytellers. Perhaps viewers felt connected to these narratives based upon their similar life stages, and their racial/ethnic identity was less important. While this finding departs from our initial hypothesis, this infers that utilizing culturally attuned interventions, like TIMB: SBC, can be instrumental across varied demographic cohorts, transcending potential cultural barriers in the discourse and understanding of mental health.

### Limitations

This paper presents invaluable insights into mental health narratives, yet several limitations warrant careful consideration. First, the potential lack of sample representativeness emerged given that certain recruitment channels attracted participants with specific characteristics, like already being interested in this study and having higher educational attainment or internet proficiency, potentially skewing the findings and restricting the generalizability. Second, using self-report measures introduces the possibility of social desirability and recall biases, challenging the validity of the data by possibly underreporting stigmatizing attitudes or inaccurately recalling past experiences. Additionally, the constrained cultural and demographic difference in the sample limits the external validity of the findings across diverse contexts and populations. The consistency of intervention delivery also presented a potential pitfall, as any variability could obscure the true impact of the intervention. Additionally, the absence of long-term follow-up assessments limited the insights into the sustained impacts and effectiveness of the intervention over time. We also recognize that our choice of a control condition that was unrelated to mental health limited our ability to examine the impact of this intervention in comparison with other contact/mental-health-education-based interventions. While the choice of control was intentionally designed to not be expected to impact stigma or mental health attitudes, this choice limited our ability to speak to the relative impact of TIMB:SBC in comparison with other related intervention models.

## 5. Conclusions

This study’s findings provide preliminary evidence that sharing mental health narratives in a virtual environment can impact viewers’ attitudes about mental health treatment and can mitigate personal stigma. Despite the limitations related to sample representativeness and measurement biases, the demonstrated impact of the intervention underscores the potential of strategic communication efforts in mitigating mental health stigmatization and enhancing empathetic, informed discourse within the community. Further, these interventions are freely accessible, providing a pathway to engage a broader community in these observed benefits.

This intervention aligns with ongoing public health efforts to improve health equity by addressing social determinants of health, such as stigma, that contribute to mental health disparities. Mental illness stigma is increasingly recognized as a critical public health issue because it not only affects individual health behaviors but also impedes the effectiveness of broader healthcare systems. By reducing stigma and improving mental health attitudes, TIMB: SBC creates an environment conducive to better health care access and utilization, which is crucial for improving health outcomes and addressing disparities. This type of intervention represents a scalable solution that can be integrated into broader mental health and public health strategies to reduce the disparities in access to care. Virtual interventions, like the TIMB: SBC platform, have become essential in the wake of the COVID-19 pandemic, which disrupted traditional health delivery systems. The integration of such culturally tailored virtual platforms into public health strategies can enhance the reach and impact of mental health services, particularly for marginalized populations. These digital interventions reduce the geographical and logistical barriers to care, offering a sustainable model for improving mental health access for underserved populations across diverse settings.

Future research endeavors should address the limitations identified in this study, augmenting the generalizability and validity through representative population-based approaches, and studying the relative impact on the outcomes of TIMB: SBC with similar interventions through comparative effectiveness trials. The explicit exploration of why and how interventions like TIMB: SBC function will be imperative to refine the intervention, enhance its efficacy, and pinpoint the most impactful elements. Future studies might benefit from qualitative analysis, diving deeper into the experience of participants to identify the differential impacts of various aspects of the intervention (e.g., personal storytelling, modality of artistic expression, or audience interaction) and mechanisms of change on stigma and mental health-related attitudes. Through the amalgamation of robust research and strategic, evidence-informed interventions, society can collectively stride toward an era where mental health is discussed, understood, and supported with empathy and knowledge without judgment.

## Figures and Tables

**Table 1:** Demographic characteristics of participants by condition.

	This Is My Brave Condition	Control Condition	Total
N	91	102	193
Age (SD)	34.53 (10.41)	32.75 (11.87)	33.59 (11.21)
Gender			
Male (%)	46 (50.5)	44 (43.1)	90 (46.6)
Female (%)	42 (2.2)	54 (52.9)	96 (49.7)
Non-binary/other (%)	2 (2.2)	4 (3.9)	6 (3.1)
Race			
African American/Black (%)	51 (56.0)	62 (60.8)	113 (58.5)
White/Caucasian (%)	40 (44.0)	40 (39.2)	80 (41.5)
Ethnicity			
Hispanic (%)	0 (0.0)	2 (2.0)	2 (1.0)
Non-Hispanic (%)	91 (100)	100 (98.0)	191 (99.0)
Sexual Orientation			
Heterosexual (%)	73 (80.2)	78 (76.5)	151 (78.2)
Bisexual (%)	10 (11.0)	15 (14.7)	25 (13.0)
Gay/lesbian/queer (%)	5 (5.5)	4 (3.9)	9 (4.7)
Questioning (%)	2 (2.2)	0 (0.0)	2 (1.0)
Other (%)	1 (1.1)	5 (4.9)	6 (3.1)
Education			
Between 9th and 12th grade but no degree (%)	0 (0.0)	1 (1.0)	1 (0.5)
High school degree (%)	14 (15.4)	19 (18.6)	33 (17.1)
Some college but no degree (%)	32 (35.2)	36 (35.3)	68 (35.2)
Associate’s degree (%)	9 (9.9)	9 (8.8)	18 (9.3)
Bachelor’s degree (%)	24 (26.4)	32 (31.4)	56 (29.0)
Graduate or professional degree (%)	12 (13.2)	5 (4.9)	17 (8.8)
Relationship Status			
Single (%)	42 (46.2)	50 (49.0)	92 (47.7)
In a relationship (%)	20 (22.0)	28 (27.5)	48 (24.9)
Married or domestic partnership (%)	26 (28.6)	17 (16.7)	43 (22.3)
Divorced (%)	2 (2.2)	7 (6.9)	9 (4.7)
Widowed (%)	1 (1.1)	0 (0.0)	1 (0.5)
Previous Diagnosis of Mental Illness			
Yes (%)	39 (42.9)	41 (40.2)	80 (41.5)
No (%)	49 (53.8)	57 (55.9)	106 (54.9)
Unsure (%)	3 (3.3)	4 (3.9)	7 (3.6)
Current Treatment			
Yes (%)	17 (18.7)	26 (25.5)	43 (23.3)
No (%)	74 (81.3)	76 (74.5)	105 (77.7)

**Table 2:** Means of dependent variables by condition and time by condition interaction results (* reflects statistically significant results).

Measure			Mean Pre/Post Differences (95%CI)				*p*-Value	Effect Size (η_p_^2^)
	Cronbach’s Alphas		TIMB Condition		Control Condition			
	Pre	Post	Pre	Post	Pre	Post		
Personal stigma	0.78	0.78	32.65	28.78	33.53	32.01	0.01 *	0.03
Perceived stigma	0.88	0.90	51.05	49.33	51.73	50.75	0.36	0.004
Discrimination	0.92	0.92	16.56	15.70	16.81	16.59	0.05 *	0.02
Perceived difference	0.93	0.97	8.33	10.41	9.16	9.19	<0.001 *	0.12
Anti-racism	0.86	0.86	35.83	36.33	35.63	35.44	0.11	0.01
Attitudes MH Tx	0.82	0.85	57.58	59.42	58.06	58.14	0.001 *	0.05
Beneficial attitudes Tx	0.67	0.70	30.02	30.91	30.13	30.01	0.01 *	0.04
Pessimistic attitudes Tx	0.77	0.79	27.56	28.52	27.93	28.07	0.02	0.03

## Data Availability

The datasets presented in this article are not readily available because of technical limitations. Requests to access the datasets should be directed to the corresponding author, Kyaien Conner.

## Appendix A

**Table A1:** *T*-test results of initial scores in Black versus White participants across variables (* reflects statistically significant results).

Measure	Black/African American (n = 113)	White (n = 80)	*p*-Values * (Two-Sided)
Personal stigma	35.12	30.95	0.01 *
Perceived stigma	52.01	50.68	0.41
Discrimination	17.33	19.83	<0.001 *
Perceived difference	7.62	9.85	<0.01 *
Anti-racism	36.31	35.10	0.19
Attitudes MH Tx	55.74	60.03	<0.001 *
Beneficial attitudes Tx	29.32	30.89	0.02 *
Pessimistic attitudes Tx	26.42	29.14	<0.001 *
